# Inkjet-Printed Temperature Sensors Characterized according to Standards

**DOI:** 10.3390/s22218145

**Published:** 2022-10-24

**Authors:** Jonas Jäger, Adrian Schwenck, Daniela Walter, André Bülau, Kerstin Gläser, André Zimmermann

**Affiliations:** 1 Hahn-Schickard, Allmandring 9b, 70569 Stuttgart, Germany; 2 Institute for Micro Integration (IFM), University of Stuttgart, Allmandring 9b, 70569 Stuttgart, Germany

**Keywords:** characterization, drift, hysteresis, inkjet, maximum error, nanoparticle, non-repeatability, temperature sensor

## Abstract

This paper describes the characterization of inkjet-printed resistive temperature sensors according to the international standard IEC 61928-2. The goal is to evaluate such sensors comprehensively, to identify important manufacturing processes, and to generate data for inkjet-printed temperature sensors according to the mentioned standard for the first time, which will enable future comparisons across different publications. Temperature sensors were printed with a silver nanoparticle ink on injection-molded parts. After printing, the sensors were sintered with different parameters to investigate their influences on the performance. Temperature sensors were characterized in a temperature range from 10 °C to 85 °C at 60% RH. It turned out that the highest tested sintering temperature of 200 °C, the longest dwell time of 24 h, and a coating with fluoropolymer resulted in the best sensor properties, which are a high temperature coefficient of resistance, low hysteresis, low non-repeatability, and low maximum error. The determined hysteresis, non-repeatability, and maximum error are below 1.4% of the full-scale output (FSO), and the temperature coefficient of resistance is 1.23–1.31 × 10^−3^ K^−1^. These results show that inkjet printing is a capable technology for the manufacturing of temperature sensors for applications up to 85 °C, such as lab-on-a-chip devices.

## 1. Introduction

Digital printing technologies, such as inkjet or aerosol jet, can be used for manufacturing functional structures. These technologies offer many advantages, such as the manufacturing of individualized products, miniaturization, short development cycles, low use of materials, or small supply chains. By printing inks with metal fillers and subsequently removing the solvents, the metal particles remain. Metal particles for digital printing are usually nanoparticles with diameters below 150 nm. Such small particle sizes enable proper printing through thin nozzles and sintering at temperatures below 200 °C [1]. By sintering the metal nanoparticles, structures with conductivities of usually 10 to 20% of the bulk materials are achieved. Such conductive structures can be used as resistive sensors, such as temperature sensors. With an increase in the temperature, the resistance of the metal structures increases due to the higher scattering of phonons [2]. This sensitivity of the electrical resistance due to changes in temperature is defined by the temperature coefficient of resistance (TCR). The currently most commonly used nanoparticles for inkjet-printed electronics are silver nanoparticles. The combination of high conductivity, low susceptibility against oxidation, and relatively low cost make silver nanoparticles a common choice over polymers, metal salts, carbon, indium tin oxide, or other metal nanoparticles, such as copper, gold, or platinum [3]. For the deposition of materials with higher viscosities, other digital printing technologies, such as electrohydrodynamic printing, piezo jet printing, or ultraprecise dispensing can be performed [4,5,6,7].

Several groups investigated inkjet-printed resistive temperature sensors. The sensor properties of the printed sensors vary due to different materials and manufacturing parameters. Bulk silver has a TCR of 3.8 × 10^−3^ K^−1^ [8]. Such a rather high TCR is desired for printed nanoparticle sensors to achieve a high sensitivity. With improved selection of materials and manufacturing parameters, researchers were able to tune the nanoparticle structures towards the property of bulk silver. The range of TCR of printed silver nanoparticle temperature sensors in the literature varies between 0.6 × 10^−3^ K^−1^ and 2.8 × 10^−3^ K^−1^ [2,9,10,11,12,13,14]. The properties of silver nanoparticle sensors are documented in Table 1. Along with the TCR, the substrate, sintering method, range of characterization, test methods, as well as the hysteresis and non-linearity of inkjet-printed silver nanoparticle temperature sensors, are listed in Table 1. The ideal sensor should have a low hysteresis and a linear behavior. The values for these properties vary between different publications and are often stated with “no hysteresis” or “linear behavior”. Hence, a good comparison cannot be performed at this point. This is a reason for one goal of this publication, which is to provide values for these properties, according to the international standard IEC 61928-2.

One important step to achieve high TCR of inkjet-printed temperature sensors is the sintering of the printed nanoparticles. High sintering temperatures increase the density and the conductivity of inkjet-printed nanoparticle structures due to higher driving forces during sintering [15]. Matthiessen’s rule states that the sensitivity of an electrical resistance to temperature only depends on the scattering of phonons [16]. Since inkjet-printed nanoparticle structures show low thicknesses and inhomogeneities, such as pores or grain boundaries, the free length of paths of electrons is lower than for bulk silver. Hence, the sensitivity (TCR) of nanoparticle structures is always lower than the sensitivity of bulk silver. However, the sensitivity increases with more densely sintered structures [2]. Increasing the sintering temperature from 150 °C to 350 °C increased the TCR from 0.6 × 10^−3^ K^−1^ to 2.8 × 10^−3^ K^−1^ in Settes’ dissertation [11]. These are the highest reported sintering temperatures and the highest reported TCR in the literature research, which shows the importance of sintering. The second highest TCR was achieved by Felba with 2.08 × 10^−3^ K^−1^. Felba printed silver nanoparticles with diameters of 4–10 nm, which is 10 to 20 times smaller than commonly used nanoparticles of around 100 nm. With such small diameters, the achievable density during sintering increases significantly, because a higher ratio of surface to volume increases the free energy in a system of smaller particles. Hence, with smaller particles, the melting temperature and also the sintering temperature decrease and nanoparticle structures can be sintered more densely at low sintering temperatures of about 200 °C [1]. This probably led to a more densely sintered structure after sintering at 250 °C and to a higher TCR than in comparable research. Other important sintering parameters to achieve high sintering densities are dwell time and heating rates. A dwell time of usually 10 to 30 min should be reached so that sintering can occur [15,17]. High heating rates are preferable, since slow heating rates can result in rounding of pores during the first phase of sintering [18]. Such an effect can lead to a reduced driving force during the following phase of sintering and higher resistances of printed nanoparticle structures [19,20].

The substrate has to be taken into account, since the different coefficients of thermal expansion of the substrate and the silver structure can influence the electrical properties [1,2]. Additionally, the substrate determines the possible sintering temperature and whether photonic sintering is possible. Another important influence on inkjet-printed temperature sensors is a protective coating. Courbat coated printed sensors with parylene, which improved the linear coefficient from 0.992 to 0.9999 and eliminated the hysteresis, while maintaining the same TCR [12]. Zikulnig investigated the influence of humidity on the performance of inkjet-printed temperature sensors. It turned out that encapsulation of printed silver structures in epoxy only resulted in minor changes in resistance due to humidity changes. For encapsulated printed sensors, the measured influence of an increase in the humidity from 20% RH to 90% RH was 0.1% at 20 °C, which equals an error of 0.69 °C. At a temperature of 60 °C, the influence of the increased humidity was 0.26%, which equals an error of 1.64 °C. Non-encapsulated sensors showed an increase of 10% due to the humidity variation, which shows that an encapsulation is a great option to reduce such cross-influences [9]. Mutee ur Rehman investigated the encapsulation of printed PEDOT:PSS temperature sensors with an Al_2_O_3_ layer. After 50 cycles between 25 °C and 90 °C at either 28–33% RH or 75% RH, the electrical resistances of non-encapsulated sensors decreased by 14.4% and 64.4%. The resistance of encapsulated sensors only decreased by 0.8% and 1.5% under the same test conditions, which is a great improvement [21].

After the manufacturing of inkjet-printed sensors, the characterization is the next important part. The characterization usually includes evaluation of the temperature coefficient of resistance, hysteresis, and linearity. More rarely characterized properties of inkjet-printed temperature sensors are drift, response time, repeatability, cross-sensitivity to bending, and humidity [2,9,10]. One difficulty concerning the characterization in the literature is that the evaluation processes were conducted differently, which makes it difficult to compare the properties of the sensors.

Table 1 also summarizes testing conditions of various articles and dissertations. The TCR was either determined by temperature steps or temperature sweeps. The drift was usually determined by the same test. In addition, the definition of drift varied within the literature. For measurement of hysteresis, the maximum variation of sweep up versus sweep down was evaluated across all temperatures or at 25 °C and set into perspective with the resistance of the printed sensor [10,14]. Testing according to standards was not described in the literature of inkjet-printed temperature sensors up to this point. Hence, one goal of this publication is to determine the properties of inkjet-printed temperature sensors according to the standard IEC 61928-2 for the first time. This will enable the comparison of such sensors across different research groups and will show if inkjet-printed sensors are able to match specifications according to standards.

**Figure 1:**
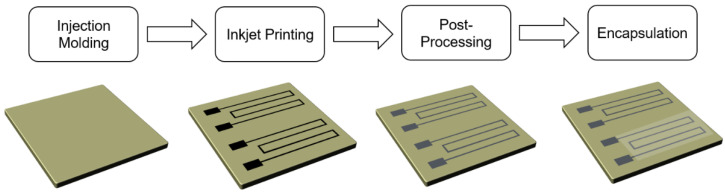
Scheme which demonstrates the processes for manufacturing of inkjet-printed temperature sensors.

The presented investigations are performed on injection-molded substrates. The injection-molded substrates used for this article are liquid crystal polymer (LCP) and cyclic olefin copolymer (COC). LCP is a substrate which is commonly used for mechatronic integrated devices (MID) and has favorable thermal properties for high reliability under thermal shock [22], while COC can be used for lab-on-a-chip applications due to its good chemical resistance and compatibility with biological samples [23]. A scheme that shows the processes for manufacturing inkjet-printed temperature sensors is displayed in Figure 1. After printing, the nanoparticle structures will be sintered and, depending on the design of experiments, encapsulated. After manufacturing, the sensors will be characterized in a climate chamber. The aimed-for temperature range for the temperature sensors is 10 to 85 °C. This covers the desired temperature of 65 °C for sensing DNA as a lab-on-a-chip device. For these devices, temperature sensors are essential for the regulation of the heating during the sensing process. The temperature range also covers 85 °C, which is an interesting peak temperature for applications in automotive interiors. The lower selected temperature of 10 °C was chosen to ensure a good regulation of the humidity level.

## 2. Materials and Methods

### 2.1. Inkjet Printing of the Temperature Sensors

Temperature sensors were manufactured by inkjet printing on injection-molded substrates. Substrate materials were cyclic olefin copolymer (COC Topas 5013 S-04 transparent from Topas Advanced Polymers, Florence, KY, USA) and liquid crystal polymer (LCP Vectra E840i LDS from Celanese, Irving, TX, USA). Prior to printing, the substrates were cleaned for 3 min in isopropyl alcohol in an ultrasonic bath and then rinsed with deionized water. Afterwards, the substrates were dried at 80 °C for 1 h. After cleaning, the substrates were treated with atmospheric plasma to achieve the desired wetting behavior during inkjet printing. The plasma system used for these trials was the FG5001 from Plasmatreat, GER, with the rotary nozzle RD1004. Plasma parameters which were not varied are distance between the nozzle and the substrate of 28.6 mm, voltage of 260 V, plasma cycle time of 100%, frequency of 21 kHz, and the use of compressed air as the working gas. The speed of the substrate table was 25 mm/s for COC and 50 mm/s for LCP.

Inkjet printing was performed on a DMP 2850 with 10 pl DMC cartridges, both from Fujifilm Dimatix, Santa Clara, CA, USA. The selected ink was a silver nanoparticle ink I30EG-1 from PV Nanocell, ISR with particle diameters d_50_ = 70 nm and d_90_ = 125 nm. The ink was printed with 1270 dpi on both substrates to enable comparable printed structures. The layer height of printed structures was measured with a white light interferometer, namely a Wyko 9100 NT from Veeco, Plainview, NY, USA. Sintering was performed thermally in a lab oven, namely a UF55 from Memmert, GER, and photonically with a PulseForge 1200 from Novacentrix, Austin, TX, USA.

Sintering varied according to the design of experiments (DoE) with parameters listed in Table 2. According to the DoE, some samples were washed in isopropyl alcohol and water for 1 min each to reduce organic residues. Some samples were coated with fluoropolymer FC-742 Certonal, Acota, Oswestry, UK. Four layers of coating were applied to ensure that the structures were fully coated. To take several manufacturing parameters into account, a L16 Taguchi design of experiment was conducted. Seven parameters with two factors were investigated in this DoE with one sample per combination. The full set of experiments is described in the Supplementary Materials. It is obvious that no statistical significance can be stated with this DoE. However, main effects can be derived and set as focus points for future investigations. The factors of the parameter sintering temperature was different for both substrates. Since LCP can be sintered at 200 °C, but not photonically, the high sintering energy for this substrate was achieved by a high sintering temperature. Since COC can be sintered photonically due to its transparency, but not thermally at 200 °C due to its lower glass transition point, the high sintering energy was achieved by photonic sintering.

### 2.2. Measurement Equipment

**Figure 2:**
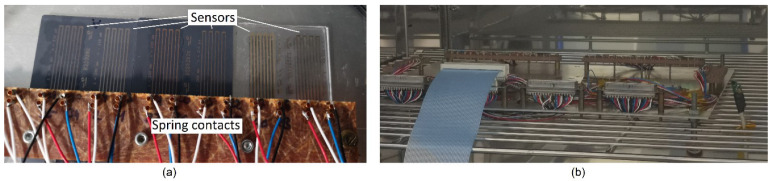
Temperature sensors connected with spring contacts (**a**) for tests in the climate chamber (**b**).

To measure the characteristic curves of the sensors, a climate chamber, namely a CV-70/350-10S from CTS GmbH, GER, was used. The reference temperature was recorded with a Sensirion SHT25 sensor. The printed sensors were connected with spring contacts, as shown in Figure 2.

**Figure 3:**
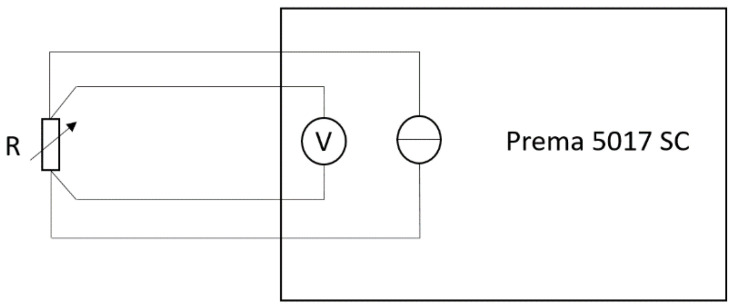
Block diagram of the measurement setup. Four-terminal sensing with separate wiring for current and voltage eliminates the influences of the measuring lines.

During the tests in the climate chamber, the electrical resistance was measured in a 4-wire mode with a digital multimeter PREMA 5017 SC with a digit resolution of up to 7.5, which was calibrated within the last year. Due to the 4-wire mode, which is displayed in Figure 3, the influences of the measuring lines are eliminated [24]. The measurement was performed in a measurement range of 300 Ω and an integration time of 4 s. The measurement error of the range, the measurement error of the calibration, the resolution due to the measurement time, and the standard deviation of each measurement point led to an expanded uncertainty between 4.4 mΩ and 5.9 mΩ, depending on the characterized sensors between 50 Ω and 100 Ω.

Scanning electron microscope (SEM) images were taken with a FEI Helios Nanolab DualBeam with an in-lens detector from Thermo Fisher, Waltham, MA, USA.

### 2.3. Measurement and Evaluation of the Characteristic Curve

**Figure 4:**
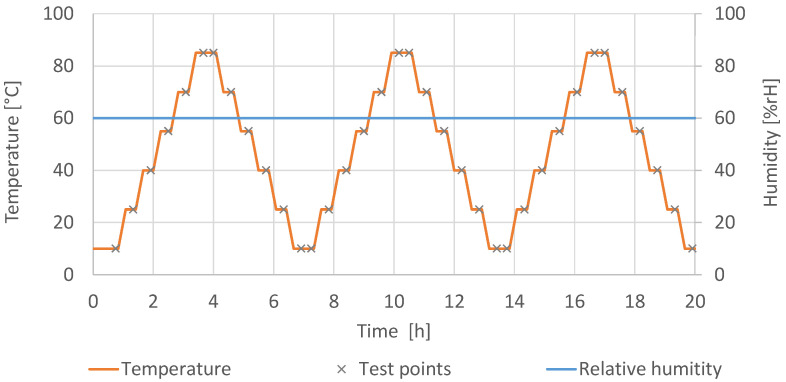
Course of temperature and relative humidity of the climate chamber. The cycles were selected according to IEC 61928-2.

The measurements of the characteristic curves were conducted according to IEC 61298-2 [25]. A preconditioning was carried out before the measurement. The sensors were cycled three times through the measurement range. To measure the characteristic curve, three measurement cycles were conducted with six test points at 0%, 20%, 40%, 60%, 80%, and 100% of the full-scale output (FSO) of the sensor. The FSO describes the difference between the output signals measured at the maximum and minimum applied temperature [26]. As the measurement range of the sensor is 10 °C to 85 °C, the full scale gives a range of 75 °C. Between the measurement points, the temperature was changed with a slope of 1 °C/min. The temperature was kept constant for 15 min before the measurement was conducted to ensure a uniform temperature distribution in the chamber and within the sensors. The humidity was kept constant at 60% RH during the measurement. This level of humidity allows us to evaluate the cross-influence of this disturbance variable. Very low values of humidity would make an evaluation of this influence difficult. Figure 4 shows the course of the temperature and the humidity during the measurement of the characteristic curve.

**Figure 5:**
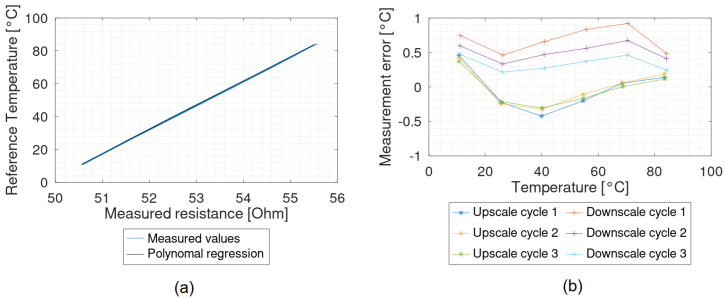
Results of a measurement; (**a**) characteristic curve of a sensor with a TCR of 1.31 × 10^−3^ K^−1^; (**b**) error curve. Both graphs were generated with the same data.

The recorded measurement values at the test points can be represented as a characteristic curve. According to the standard IEC61298-2, the calibration was conducted with the second upscale cycle. For the temperature sensors, a linear fit was selected (Figure 5a). The reciprocal of the slope of the regression, divided by the resistance of the sensor, represents the TCR of the sensor.

The measured values were calibrated with the regression curve and the error curve was generated (Figure 5b). Based on the error curve, the values for the non-repeatability, the hysteresis and the maximum measured error were determined according to the standard. Hysteresis is the maximum difference between a measured value in a consecutive up- and downscale cycle at the same reference temperature. Non-repeatability, on the other hand, is the maximum deviation at the same measurement point when approaching from the same direction. The maximum measured error is the maximum absolute deviation from the nominal value. The resulting values are converted to percent of the FSO [27].

The start-up drift was also tested according to the standard IEC61298-2. After subjecting the sensors to ambient environmental conditions for more than 12 h, the sensors were put in a pre-heated climate chamber. The temperature was set to 77.5 °C, which is equivalent to 90% of the maximum input signal of 85 °C. The resistance reading was then recorded for another 4 h. The time for the sensor to reach and remain in a limit of 1% FSO is defined as the start-up drift.

## 3. Results

### 3.1. Printing

**Figure 6:**
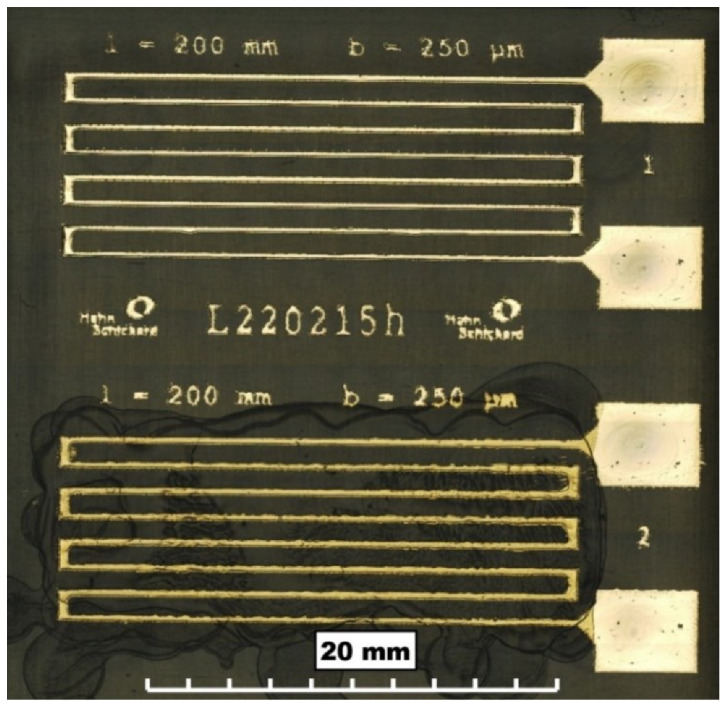
Inkjet-printed temperature sensor (1) without coating and (2) with coating.

Silver nanoparticle structures were successfully printed on LCP and COC. The wetting after plasma pre-treatment was sufficient to print sensor structures, as displayed in Figure 6 After printing, sintering of the nanoparticle structures was performed to create conductive paths. With a length of 200 mm, measured median widths of 340 µm, and median heights of 1.2 µm, the specific electrical resistance of the printed structures was calculated. The lowest specific electrical resistances were 8.5 µΩ∙cm, achieved by photonic sintering. The lowest specific electrical resistances achieved by thermal sintering were 10.6 µΩ∙cm.

**Figure 7:**
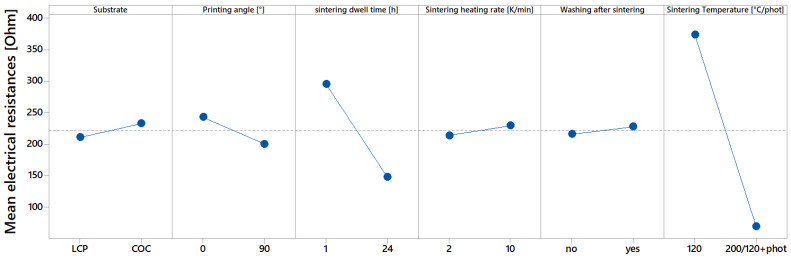
Main effect diagram, which indicates influences of manufacturing parameters on electrical conductivity.

The influences of the manufacturing parameters on the electrical resistance of the printed sensors are described in a main effect diagram in Figure 7. For each manufacturing parameter, two sensors were sintered, and the mean electrical resistance was used for calculation of the main effects. For two sensor pairs, the standard deviation of the resistance was off by more than 10%, which indicates printing inhomogeneity on the sensor with the higher resistance. Thus, only the resistance of the sensor with the lower resistance was selected for calculation. The main effect diagram reveals that some manufacturing parameters seem to strongly affect the electrical resistance of the printed sensors. The sintering temperature seems to have an especially prominent effect on the electrical resistance. The second biggest influencing factor on the electrical resistance seems to be the dwell time. Other parameters, such as the substrate, the angle between the printed meander and the print-head’s direction of movement, the heating rate, and washing in isopropyl alcohol and deionized water, only seem to have minor influences, compared to the sintering parameters.

### 3.2. Characteristic Curve

**Figure 8:**
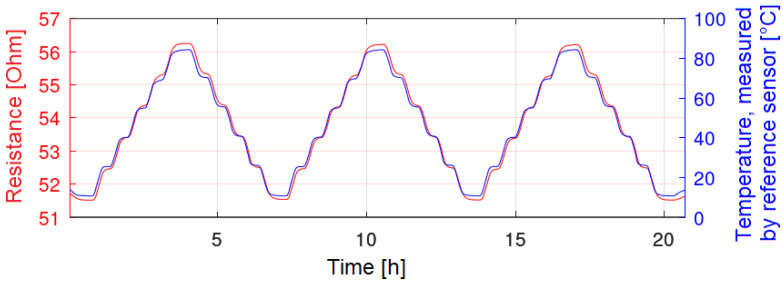
Inkjet-printed temperature sensor (red), which follows the temperatures steps well.

After the evaluation of the printing results and the electrical resistances, the same sensors were characterized in a climate chamber by temperature cycles. The measurement results of these tests are displayed as a function of the electrical resistance over time (Figure 8, red curve). The temperature, tracked by a SHT25, is reflected by the blue curve.

**Figure 9:**
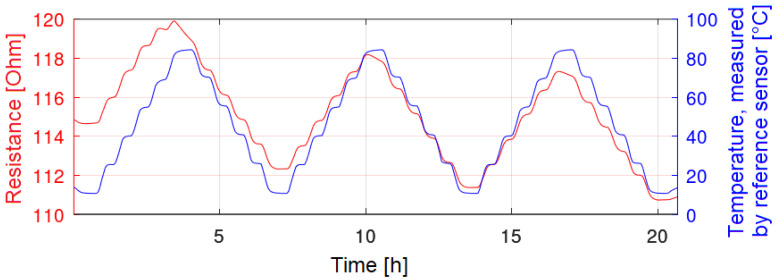
Inkjet-printed temperature sensor, which shows high hysteresis and non-repeatability.

Figure 8 represents a good sensor. It can be seen that the hysteresis and the non-repeatability seem to be low, and that the sensor signal follows the set temperature well. Figure 9, on the other hand, represents a worse sensor. Especially at elevated temperatures, such as 85 °C, a decrease in the electrical resistance can be observed. Hence, the non-repeatability and the hysteresis for these sensors are high. The temperature coefficient of resistance was calculated for sensors, which showed curves similar to Figure 8. Table 3 shows the calculated TCR for such sensors. Depending on the manufacturing conditions, TCRs of up to 1.41 × 10^−3^ K^−1^ were achieved. This table already shows that only sensors sintered either at 200 °C or sintered photonically have good properties and could be characterized sufficiently. On the other hand, sensors only sintered at 120 °C on both substrates had worse properties and their TCRs are not displayed in Table 3.

**Figure 10:**
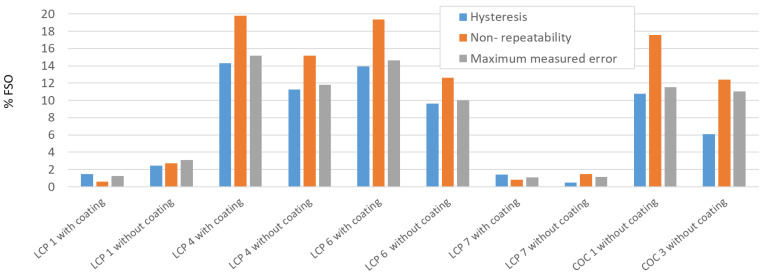
Measurement results of the characteristic curve, showing hysteresis, non-repeatability, and maximum measured error.

Figure 10 illustrates the measurement results regarding hysteresis, non-repeatability, and maximum measured error for the best tested sensors, presented in Table 3. These measurements show that especially LCP 1 and LCP 7 achieved rather good results. Indeed, LCP 1 and LCP 7 were the only sensors, sintered at 200 °C for 24 h. Once more, this indicates that the sintering process is important to achieve good temperature sensor properties.

**Figure 11:**
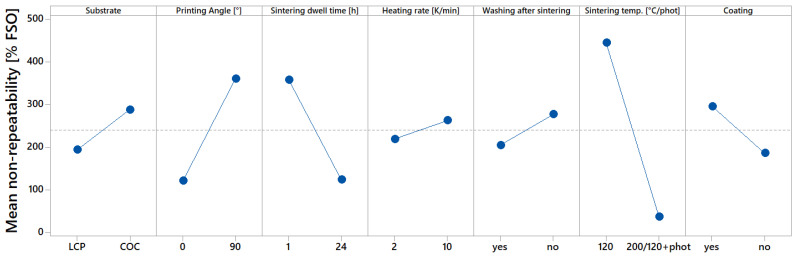
Main effect diagram, which indicates influences of manufacturing parameters on non-repeatability [% FSO].

Figure 11 further evaluates the influence of manufacturing parameters on the sensor properties. Representative of the quality of the characterized sensors, the influence on the non-repeatability is displayed in this main effect diagram. For this figure, all 32 manufactured sensors were taken into account. It can be seen that rather low non-repeatability can only be achieved with high sintering temperatures or photonic sintering. It can also be seen that extremely high non-repeatability was observed for all sensors, which were not sintered at 200 °C or photonically.

**Figure 12:**
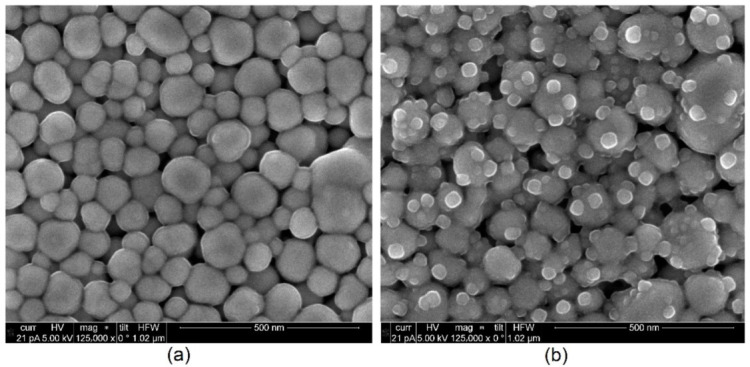
SEM images of silver nanoparticles, sintered with different energies. (**a**) 120 °C, 1 h with a heating rate of 2 K/min. (**b**) 200 °C, 24 h with a heating rate of 2 K/min.

The SEM images in Figure 12 show the influence of sintering temperature on the nanoparticle network. Sensors sintered at 120 °C only show connectivity between nanoparticles, whereas sensors sintered at 200 °C show sintering necks. This reflects the higher sintering degree and explains the higher conductivity of the structures sintered at 200 °C.

### 3.3. Start-Up Drift

**Figure 13:**
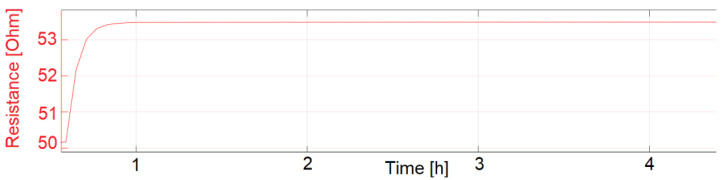
LCP 7 (with coating) start-up drift.

**Figure 14:**
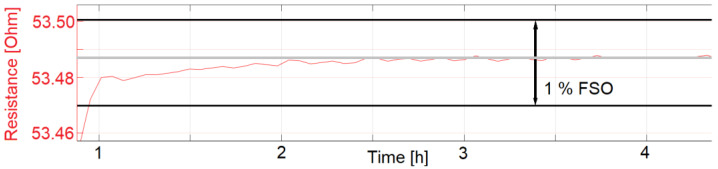
LCP 7 (with coating) start-up drift, zoomed to 1% FSO.

**Figure 15:**
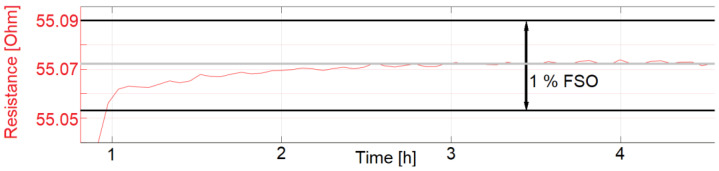
LCP 1 (with coating) start-up drift, zoomed to 1% FSO.

For measurement of the start-up drift, inkjet-printed temperature sensors were first stored at a room temperature of 23 °C with humidity at 57% RH for 18 h. The sensors were then put in the pre-heated climate chamber at 77.5 °C and 60% RH. For an exact measurement of the start-up drift, some cross-influences need to be taken into account. First, the chamber cooled to around 65 °C and 30% RH during the opening and set up of the test. A SHT25 measured that the chamber needed around 10 min to reach the desired temperature and humidity. Other influences are that the sensors were printed on 1.4 mm thick polymer substrates, which were mounted on a 1 mm thick steel plate. Both the plate and the substrates need time to reach the set temperature. With the described influences, the start-up drift was measured to be 21.3 and 24.7 min for sensors sintered at 200 °C for 24 h and subsequently coated with fluoropolymer. The start-up drift is defined as the time the temperature sensors need until their resistance reading is within 1% of the desired resistance. The measurements which were used to calculate the start-up drift are displayed as graphs in Figure 13, Figure 14 and Figure 15.

**Figure 16:**
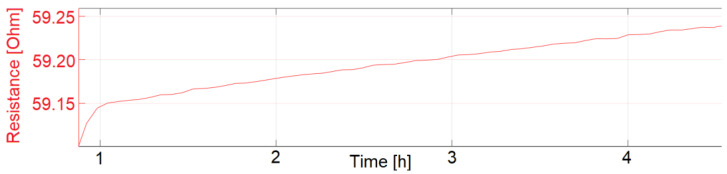
LCP 1 (without coating) start-up drift. Sensor signal drifts with ongoing exposure to 77.5 °C and 60% RH.

**Figure 17:**
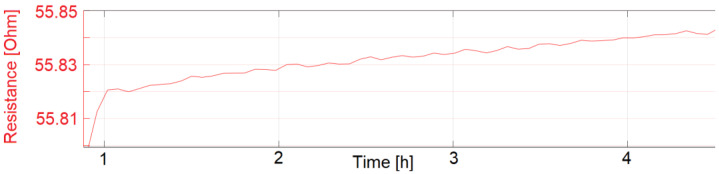
LCP 7 (without coating) start-up drift. Sensor signal drifts with ongoing exposure to 77.5 °C and 60% RH.

The start-up drift was only calculated for the sensors presented in Figure 13, Figure 14 and Figure 15. Sensors with identical manufacturing parameters, but without a coating with fluoropolymer, did not show the desired start-up behavior. Instead, a drift in the electrical resistance could be observed for these sensors, shown in Figure 16 and Figure 17. A drift of the electrical resistance was also observed for other tested sensors, such as LCP 6, COC 1, and COC 3.

## 4. Discussion

This publication provides an analysis of the capabilities of inkjet-printed temperature sensors. High sintering temperature, long dwell time, or high energies due to photonic sintering led to improved sensor properties, such as the temperature coefficient of resistance, hysteresis, non-repeatability, and maximum error. The best values for hysteresis, non-repeatability, and maximum error of 0.6% to 1.4% FSO were achieved with the highest investigated sintering temperature of 200 °C and the longest dwell time of 24 h. These sintering conditions led to further diffusion processes and to rather densely sintered structures with improved sensor properties. Lower dwell times of 1 h at 200 °C led to higher hysteresis, non-repeatability, and maximum error of up to 20% FSO. Lower sintering temperatures of 120 °C for either 1 h or 24 h led to poorly sintered structures and poor sensor properties. None of the sensors sintered at 120 °C were able to achieve a low hysteresis, a low non-repeatability, or a low maximum error. Hence, one result of these investigations is that the sintering is one of the most important manufacturing processes for inkjet-printed temperature sensors. Since the influence of the sintering energy was extremely high, the influence of other tested manufacturing parameters, such as the printing direction, the heating rate, or the treatment in water and isopropyl alcohol could not be proven. The highest TCR of 1.41 × 10^−3^ K^−1^ was achieved by photonic sintering. However, the properties for hysteresis, non-repeatability, and maximum error were between 6 and 12% for this sensor. This shows that the printed sensor should always be analyzed in terms of TCR as well as hysteresis, non-repeatability, and maximum error. For an explanation of the highest TCR, achieved by photonic sintering, but lower values for hysteresis, non-repeatability, and maximum error, statistical analysis in future research should be performed.

Besides the sintering, a coating with fluoropolymer improved the start-up drift and a stable resistance could be measured. This was not the case for non-coated sensors. We propose that future tests should only be performed with high sintering energies, comparable to sintering at 200 °C for 24 h and with a coating of the sintered structures, in order to achieve the best possible sensor properties. Once these parameters are set, other manufacturing parameters, such as the printing direction, can be evaluated as well.

Room for improvement can be seen in two areas of inkjet-printed temperature sensors. The first possible improvement can be achieved regarding the electrical properties. Higher sintering degrees should lead to even better electrical properties of the printed sensors. Such higher sintering degrees can be achieved by higher sintering temperatures or alternative sintering strategies, such as light-based sintering technologies [28]. For photonic sintering, a two-step sintering is recommended by Kang et al. to achieve higher sintering degrees and lower long-term drift of the electrical resistance [29]. Another possibility to enhance the sintering degree is the size of the nanoparticles. Smaller nanoparticles enable a higher driving force which ultimately leads to higher sintering degrees [1]. A second improvement is possible for reduction in time and costs. Sintering at 200 °C for 24 h is a long and expensive process. Photonic sintering of inkjet-printed structures will reduce the process time and the corresponding costs drastically. Additionally, this will enable the manufacturing of temperature sensors on cheaper substrates, which cannot be operated at high temperatures [30]. With the reduction in costs of the sintering and the use of low-cost substrates, inkjet printing will be an even more interesting manufacturing technology. Hence, future research should address those two issues. Along with the low use of ink during the inkjet-process, the described process chain enables a cost- and resource-efficient manufacturing of sensors. Since inkjet printing is also a digital process, a wide variety of substrates can be functionalized with various sensor layouts.

Another goal of this publication was to establish a transparent and comprehensive way to characterize inkjet-printed temperature sensors. The performed cyclic temperature tests and the test of the start-up drift seem to be good methods for such a characterization. The determination of a difference between the two best coated and uncoated sensors was only possible by testing the start-up drift, but not by testing with temperature steps with much shorter cycle times. Hence, we propose that future tests should be performed according to the standards presented here. For future research, the evaluation of long-term drift will be of interest as well. A full factorial DoE with high sintering energies and a larger sample size should be performed to further evaluate the influences on the properties of inkjet-printed temperature sensors. With this future research, the good results of the screening presented here can be proven. With such tests according to standards, comparisons across different researchers are possible, and the best manufacturing parameters of inkjet-printed temperature sensors can be identified.

## 5. Conclusions

A quantity of 32 sensors was manufactured with different parameters. The most important manufacturing parameters, as well as the characterized properties of the two best inkjet-printed temperature sensors, are summarized in Table 4.

With a wide field of varied manufacturing parameters, the goal of this publication is not to statistically prove the influence of all manufacturing parameters, but to analyze if inkjet-printed temperature sensors are able to meet some specifications if they are tested according to standards. Furthermore, one of our goals is to distinguish if there are certain parameters, which need to be set correctly to manufacture inkjet-printed temperature sensors. In this research, a Taguchi DoE was performed to consider the influence of different manufacturing parameters on the electrical properties of inkjet-printed temperature sensors. The sensitivity of the printed temperature sensors was then tested with a cyclic temperature step-test according to standards of IEC 61928-2 at a temperature range of 10 °C to 85 °C at 60% RH.

Sensors that were sintered either photonically or thermally at 200 °C achieved the most stable values in the tested temperature range. Sintering inkjet-printed silver nanoparticle structures at only 120 °C was not sufficient to establish stable temperature sensors regarding TCR, hysteresis, non-repeatability, and maximum error. The evaluation of hysteresis, non-repeatability, and maximum error shows that all four sensors, sintered at the highest temperature of 200 °C for the longest dwell time of 24 h, established the best values for these properties. This shows that it is extremely important to manufacture inkjet-printed temperature sensors with sufficiently high sintering energies. After the characterization of TCR, hysteresis, non-repeatability, and maximum error, a second test was performed to determine the start-up drift according to the standards of IEC 61928-2. Two of the tested sensors were able to reach a stable resistance at the set temperature of 77.5 °C and a humidity of 60% RH. Those were the only sensors which were sintered at 200 °C for 24 h and coated with a fluoropolymer. Their properties are listed in Table 4. It has to be noted that the heating of the polymer substrate and the test bench have to be taken into account for the analysis of the start-up drift time. Two other sensors, which did not deliver a stable resistance reading, were manufactured likewise, but not coated with fluoropolymer. This shows that sintering and coating seem to be two very important manufacturing parameters for inkjet-printed temperature sensors. Both sensors presented in Table 4 were manufactured with different printing directions, sintered with different heating rates, and either washed with isopropyl alcohol and water or not. These processes do not seem to have an equally high influence as sintering and coating.

We suggest that inkjet-printed temperature sensors should be sintered with high energies and coated with a protective layer to achieve the best possible sensor properties. We also suggest that the evaluation of future inkjet-printed temperature sensors should be performed according to these standards, in order to guarantee an appropriate comparison across different researches.

## Figures and Tables

**Table 1:** Inkjet-printed resistive temperature sensors based on silver nanoparticles. The presented properties of the sensors were not tested according to a standard.

Publication	Substrate	Curing	Range of Characterization	Test Methods	Calculated Properties	Sensitivity [10^−3^ × K^−1^]	Hysteresis	Non-Linearity
Zikulnig 2021 [9]	Paper	UV/Vis (PulseForge)	−20–60 °C	1 cycle temperature steps: 20|40|60|40|20|0|−20|0|20 °C	TCR, response time, hysteresis, linearity	1.58–1.71	“no”	“linear”
Zikulnig 2019 [10]	Paper	UV/Vis (PulseForge)	20–80 °C	Temperature sweep with 2 K/min from 20 °C to 80 °C and back at 0% RH. 15 cycles	TCR, linearity, baseline drift, hysteresis	1.63–1.71	0.02–0.19% compared to full resistance	“linear”
Polzinger 2017 [2]	Polyimide	250 °C	10–50 °C	1 cycle temperature steps with dwell time of 60 min per step at 55% RH: 10|20|30|40|50|40|30|20|10	TCR	1.61	0.029 ± 0.008 °C	0.023 ± 0.025 °C
				30 cycles temperature steps with dwell time of 60 min per step at 55% RH: 10|30|50|30|10	Hysteresis, non-linearity, repeatability, drift			
Sette 2014 [11]	Silicon	150–350 °C	25–75 °C	Temperature steps with dwell time of 4 min per step: 25|45|55|65|75|65|55|45|35|25. TCR measurement on decreasing temperature	TCR, linearity	0.6–2.8	No info	Linearity R^2^ = 0.99
Molina-Lopez 2013 [14]	PET	150 °C	−10–60 °C	Temperature sweep from −10 °C to 60 °C and back at 40% RH.	TCR, hysteresis, linearity	0.65	<1% compared to resistance	Linearity R^2^ > 0.999
Courbat 2011 [12]	Paper	150 °C	−20–60 °C	Temperature variation between −20 °C and 60 °C at 30% RH.	TCR, Hysteresis, linearity	1.1	“No“ due to coating	Linearity R^2^ = 0.992–0.9999 (coating)
Felba 2009 [13]	Glass	250 °C	30–180 °C	Resistance measurement at 6 different temperatures: 30, 60, 90, 120, 150, 180 °C	TCR	2.08	No info	No info

**Table 2:** The DOE with seven parameters and two factors. Those parameters were investigated in a Taguchi screening design of experiment.

Parameter	Factor 1	Factor 2
Substrate	COC	LCP
Sintering temperature	120 °C	200 °C (for LCP) and 120 °C and photonic sintering 1.1 J/cm^2^ (for COC)
Sintering time	1 h	24 h
Heating rate	2 K/min	10 K/min
Printing angle	0°	90°
Washing after printing	No	1 min isopropyl and 1 min deionized water
Coating with fluoropolymer	No	Yes

**Table 3:** Temperature coefficient of resistance (TCR) for inkjet-printed temperature sensors on LCP and COC substrates.

Number	Coating	Sintering	TCR [10^−3^ K^−1^]
LCP 1	Yes	200 °C, 24 h	1.31
LCP 1	No	200 °C, 24 h	1.29
LCP 4	Yes	200 °C, 1 h	0.85
LCP 4	No	200 °C, 1 h	0.89
LCP 6	Yes	200 °C, 1 h	0.84
LCP 6	No	200 °C, 1 h	0.89
LCP 7	Yes	200 °C, 24 h	1.23
LCP 7	No	200 °C, 24 h	1.20
COC 1	No	120 °C, 24 h and photonic	0.93
COC 3	No	120 °C, 1 h and photonic	1.41

**Table 4:** Important properties of the two best presented printed sensors.

Label	Sintering	Coating	Ω at 20 °C	TCR [10^−3^ K^−1^]	Hysteresis [% FSO]	Non-Repeatability [% FSO]	Maximum Error [% FSO]	Start-Up Drift [min]
LCP 1	200 °C, 24 h	Yes (Fluoropolymer)	52	1.31	1.4	0.6	1.2	24.7
LCP 7	200 °C, 24 h	Yes (Fluoropolymer)	50	1.23	1.4	0.8	1.1	21.3

## Data Availability

All data used are shown in the text and the Supplementary Materials. Raw data are available on request.

## Supplementary Materials

The following supporting information can be downloaded at: https://www.mdpi.com/article/10.3390/s22218145/s1, Table S1: DoE with varying manufacturing parameters; Table S2: Properties of all 32 sensors.
