# Supplementary material for: Inkjet-Printed Temperature Sensors Characterized according to Standards
Source: Sensors (Basel). 2022 Oct 24;22(21):8145. doi: 10.3390/s22218145 (PMC9654304; doi:10.3390/s22218145)
Supplement: Supplementary file 1 [file sensors-22-08145-s001.zip › sensors-1953747-supplementary.pdf]

sensors

MDPI

Supplementary Materials

Inkjet-Printed Temperature Sensors Characterized According to Standards

Table S1. DoE with varying manufacturing parameters.

Substrate	Printing Angle [°]	Sintering dwell time [h]	Heating rate [K/min]	Washing after sintering	Sintering temp. [°C/phot]	Coating	Name
LCP	0	1	2	yes	120	yes	LCP 2 with coating
LCP	0	1	2	yes	120	no	LCP 2 without coating
LCP	0	1	10	yes	200/120+phot	yes	LCP 4 with coating
LCP	0	1	10	yes	200/120+phot	no	LCP 4 without coating
LCP	0	24	2	no	120	yes	LCP 5 with coating
LCP	0	24	2	no	120	no	LCP 5 without coating
LCP	0	24	10	no	200/120+phot	yes	LCP 7 with coating
LCP	0	24	10	no	200/120+phot	no	LCP 7 without coating
LCP	90	1	2	no	200/120+phot	yes	LCP 6 with coating
LCP	90	1	2	no	200/120+phot	no	LCP 6 without coating
LCP	90	1	10	no	120	yes	LCP 8 with coating
LCP	90	1	10	no	120	no	LCP 8 without coating
LCP	90	24	2	yes	200/120+phot	yes	LCP 1 with coating
LCP	90	24	2	yes	200/120+phot	no	LCP 1 without coating
LCP	90	24	10	yes	120	yes	LCP 3 with coating
LCP	90	24	10	yes	120	no	LCP 3 without coating
COC	0	1	2	no	200/120+phot	yes	COC 6 with coating
COC	0	1	2	no	200/120+phot	no	COC 6 without coating
COC	0	1	10	no	120	yes	COC 8 with coating
COC	0	1	10	no	120	no	COC 8 without coating
COC	0	24	2	yes	200/120+phot	yes	COC 1 with coating
COC	0	24	2	yes	200/120+phot	no	COC 1 without coating
COC	0	24	10	yes	120	yes	COC 2 with coating
COC	0	24	10	yes	120	no	COC 2 without coating
COC	90	1	2	yes	120	yes	COC 7 with coating
COC	90	1	2	yes	120	no	COC 7 without coating
COC	90	1	10	yes	200/120+phot	yes	COC 3 with coating
COC	90	1	10	yes	200/120+phot	no	COC 3 without coating
COC	90	24	2	no	120	yes	COC 5 with coating
COC	90	24	2	no	120	no	COC 5 without coating
COC	90	24	10	no	200/120+phot	yes	COC 4 with coating
COC	90	24	10	no	200/120+phot	no	COC 4 without coating

Table S2. Properties of all 32 sensors.

Sample No.	Hysteresis	Non-repeatability	Maximum measured error	Electrical resistance at room temperature	TCR [10^-3 K^-1]
R220202y with coating	1,4	0,6	1,2	52,00	1,31
R220202y without coating	2,5	2,7	3,1	55,00	1,29
R220202B with coating	57,2	84,0	60,6	482,00	0,16
R220202B without coating	11,1	16,5	12,5	515,00	0,15
R220202z with coating	278,8	368,5	285,4	250,00	0,36
R220202z without coating	175,3	238,2	179,3	259,00	0,36
R220202C with coating	14,3	19,8	15,2	94,00	0,85
R220202C without coating	11,2	15,2	11,8	93,00	0,89
R220202D with coating	90,1	123,0	92,3	257,00	0,30
R220202D without coating	41,6	57,6	43,6	253,00	0,32
R220202A with coating	13,9	19,3	14,6	102,00	0,84
R220202A without coating	9,6	12,6	10,0	93,00	0,89
R220202F with coating	1,4	0,8	1,1	51,00	1,21
R220202F without coating	0,5	1,4	1,2	53,00	1,20
R220202v with coating	1134,5	1248,1	1249,8	1200,00	-0,65
R220202v without coating	668,1	878,1	681,4	280,00	0,52
L220215h with coating	31,2	48,3	33,2	89,00	0,80
L220215h without coating	10,7	17,5	11,5	83,00	0,93
L220215i with coating	133,9	201,5	139,6	258,00	0,27
L220215i without coating	52,0	81,5	59,4	244,00	0,33
L220215c with coating	20,0	32,0	21,7	41,00	1,39
L220215c without coating	6,1	12,4	11,0	42,00	1,41
L220215e with coating	61,4	95,1	65,4	89,00	0,77
L220215e without coating	16,5	25,6	17,6	93,00	0,86
L220215d with coating	283,9	384,1	295,6	139,00	0,40
L220215d without coating	213,4	319,1	225,0	216,00	0,33
L220202-1 with coating	151,1	201,2	157,9	39,00	1,22
L220202-1 without coating	43,6	80,8	64,7	40,00	1,20
L220215b with coating	1054,3	1214,7	1071,0	545,00	0,18
L220215b without coating	713,1	913,3	732,7	543,00	0,19
L220202-2 with coating	616,4	680,0	642,3	617,00	0,94
L220202-2 without coating	218,0	294,0	259,0	724,00	0,90
